# Myelin Basic Protein-primed T Helper 2 Cells Suppress Microglial Activation via AlphaVBeta3 Integrin: Implications for Multiple Sclerosis

**DOI:** 10.4172/2155-9899.1000158

**Published:** 2013-08-12

**Authors:** Avik Roy, Kalipada Pahan

**Affiliations:** 1 Department of Neurological Sciences, Rush University Medical Center, Chicago, IL, USA; 2 Division of Research and Development, Jesse Brown Veterans Affairs Medical Center, 820 South Damen Avenue, Chicago, USA

**Keywords:** Multiple sclerosis, Microglial activation, T helper 2 cells, Inflammation

## Abstract

Multiple sclerosis (MS) is the most common autoimmune demyelinating disease in human and T helper type 2 (Th2) cells have been shown to be beneficial for this disease. However, mechanisms by which Th2 cells ameliorate disease in MS are poorly understood. Microglial activation plays an important role in the pathogenesis of MS and other neurodegenerative disorders. Here, we delineate that Th2 cells are capable of suppressing microglial activation via cell-to-cell contact. After polarization of MBP-primed Th1 cells to Th2 by gemfibrozil and other drugs, we observed that MBP-primed Th2 cells dose dependently inhibited the production of interleukin-1β (IL-1β) and nitric oxide (NO) in LPS-stimulated microglia via cell-to-cell contact. Similarly, Th2 cells also suppressed the microglial inflammatory response in the presence of different pathological stimuli of Alzheimer’s disease (AD), Parkinson’s disease (PD), and HIV associated dementia (HAD). Interestingly, Th2 cells expressed higher levels of alphaV (αV) and beta3 (β3) integrins as compared to Th1 cells, and functional blocking antibodies against αV and β3 integrins impaired the ability of Th2 cells to suppress microglial activation. Furthermore, we demonstrate that microglia expressed the beta subunit of PDGF receptor (PDGFRβ) and that neutralization of PDGFRβ abrogated the ability of Th2 cells to suppress microglial inflammation. Activation of microglial cAMP response element-binding (CREB) by Th2 cells, suppression of CREB activation by neutralization of either αV and β3 integrins on Th2 cells or PDGFRβ on microglia, abrogation of anti-inflammatory activity of Th2 cells by siRNA knockdown of microglial CREB, highlights the importance of αVβ3 and PDGFRβ in guiding the anti-inflammatory activity of Th2 cells via activation of CREB, which may be responsible for beneficial effect of Th2 cells in MS and other related disorders.

## Introduction

Multiple sclerosis (MS) is the most common human demyelinating disease in the CNS characterized by the demyelination as a result of a T cell-mediated autoimmune response [1]. Autoimmune T cells cross the blood brain barrier (BBB), infiltrate the CNS parenchyma, and initiate an inflammatory response [2]. The inflammatory role of autoimmune T cell stems from its crosstalk with microglia, a resident macrophage of the brain [3]. The increased interaction between T cell and microglia leads to produce a series of proinflammatory molecules in microglia that directly or indirectly induce oligodendroglial demyelination and neuronal loss [1,4–6]. In support of the mechanism, glia-derived cytokines including IL-1β and NO have been shown directly to induce caspases [7], mitochondrial-oxidative stress [8], and other inflammatory reactions [9] in both oligodendrocytes as well as in neuron. Therefore, suppressing microglial activation is an important aspect of research in developing new therapeutic strategy in MS.

So far, several molecules have been reported to suppress glial inflammation in the CNS. Inhibitors of different protein kinases [10,11] including MAP kinase inhibitors RWJ67657 [12], SB203580 [13], and Go6976 [14]; protein kinase C inhibitors, and MLK inhibitors have been shown to inhibit the mRNA expression of and IL-β. Recently, we have shown that carboxy- PTIO, a well-known scavenger of NO [15] and NBD peptide, a NFkB inhibitory-peptide [16,17] significantly attenuated glial inflammation. Apart from these inhibitors, there are also other drugs, which are commonly used for some other disease have been shown to reduce neuroinflammation. For an example, lovastatin, HMG-CoA reductase inhibitor [18]; sodium phenyl acetate (NaPA), a known urea cycle disorder drug [19]; and sodium phenylbutyrate, a prodrug of NaPA [20] inhibited the glia-derived cytokine production. Here we first time highlight the anti-inflammatory role of Th2 cells on account of its ability to contact with microglial cells.

In our previous study we have shown that MBP-primed Th2 cells are capable of inducing neurotrophins in microglial cells via cell-to-cell contact mechanism [3]. Here we have first shown that MBP-primed Th1 cells are capable of inducing the expression of IL-1β and iNOS in microglia via similar T cell-microglia contact and once polarized towards Th2 phenotype, these autoimmune T cells fail to produce the proinflammatory mediators in glial cells via contact mechanism. Interestingly, these T cells efficiently employ similar mechanism to inhibit the glial inflammation triggered by other disease state stimuli like LPS, IL-1β, polyIC, amyloid-β, and gp120 implicating its wide spectrum therapeutic significance. While studying the possible contact molecules in T cell plasma membrane, we observed that MBP-primed Th2 cells express wide range of α and β integrins [21] and among these integrins αV and β3 integrins are upregulated in Th2 condition. The crosstalk of αVβ3 in MBP-primed Th2 cells and PDGFR-β in microglia triggers the activation of CREB to reduce the production of proinflammatory cytokines and iNOS in microglia. Taken together, our present work suggests a novel and appealing anti-inflammatory role of αVβ3 integrins in the context of CNS disorders.

## Materials and Methods

### Reagents

Fetal bovine serum (FBS), Hanks’ balanced salt solution (HBSS), DMEM/F-12, RPMI 1640, L-glutamine, and β-mercaptoethanol were from Mediatech. Mouse recombinant interferon-was obtained from R&D. Assay systems for IL-1β and TNF-α were purchased from BD Biosciences. Bovine myelin basic protein was purchased from Invitrogen. Gemfibrozil and Poly(IC) were purchased from Sigma and HIV-1 coat protein gp120 was obtained from US Biological.

### Isolation and purification of antigen-primed T cells

Female SJL/J mice were immunized subcutaneously with 400 μg of bovine MBP and 60 μg of *Mycobacterium tuberculosis* (H37RA, Invitrogen) in Incomplete Freund’s Adjuvant (Calbiochem). Lymph nodes were collected from these mice, and single cell suspension was prepared in RPMI 1640 medium containing 10% FBS, 2 mM L-glutamine, 50 μM-mercaptoethanol, 100 units/ml penicillin, and 100 μg/ml streptomycin. Cells were cultured at a concentration of 4–5×10^6^ cells/ml in six-well plates. Cells isolated from MBP-immunized mice were incubated with 50 μg/ml MBP for 4 days. The non-adherent cells were collected and passed through the nylon wool column preincubated for a period of 30 min with RPMI 1640 supplemented with 10% FBS at 37°C, 5% CO_2_. The first 15–20-ml eluant was collected, centrifuged at 500×g, and resuspended in RPMI 1640 medium-FBS. Viability and purity of the cells were checked by trypan blue exclusion and FACS analysis, respectively. Approximately 98% cells were found as CD3-positive T cells [5]. These T cell populations were used to stimulate microglial cells.

### Isolation of mouse primary microglia

Microglial cells were isolated from mixed glial cultures according to the procedure of Guilian and Baker [5]. On days 7–9, the mixed glial cultures were washed three times with DMEM/F-12 and subjected to a shake at 240 rpm for 2 h at 37°C on a rotary shaker. The floating cells were washed and seeded onto plastic tissue culture flasks and incubated at 37°C for 2 h. The attached cells were removed by trypsinization and seeded onto new plates for further studies. Approximately, 90–95% of this preparation was found to be positive for Mac-1 surface antigen. For the induction of TNF-α production, cells were stimulated with MBP-primed T cells in serum-free DMEM/F-12. Mouse BV-2 microglial cells (a kind gift from Virginia Bocchini of University of Perugia) were also maintained and induced with different stimuli as indicated above.

### Stimulation of mouse BV-2 microglial cells and primary microglia by MBP-primed T cells

Microglial cells were stimulated with different concentrations of MBP-primed T cells under serum-free condition. After 1 hr of incubation, culture dishes were shaken and washed thrice with HBSS to lower the concentration of T cells. Earlier, by fluorescence-activated cell sorting analysis of adherent microglial cells using fluorescein isothiocyanate-labeled anti-CD3 antibodies, we demonstrated that more than 80% T cells were removed from microglial cells by this procedure [5]. Then microglial cells were incubated in serum-free media for different periods of time depending on experiments.

### Semiquantitative RT-PCR analysis

Total RNA was isolated from BV-2 microglial cells, primary microglia using Ultraspec-II RNA reagent (Biotecx Laboratories, Inc.) following manufacturer’s protocol. To remove any contaminating genomic DNA, total RNA was digested with DNase. Semiquantitative RT-PCR was carried out as described earlier [15] using a RT-PCR kit from Clontech. Briefly, 1 μg of total RNA was reverse-transcribed using oligo(dT) 12–18 as primer and MMLV reverse transcriptase (Clontech) in a 20 μl reaction mixture. The resulting cDNA was appropriately diluted, and diluted cDNA was amplified using titanium Taq DNA polymerase and the following primers. Amplified products were electrophoresed on a 1.8% agarose gel and visualized by ethidium bromide staining: iNOS, sense: 5′-CTC CTT CAA AGA GGC AAA AAT A -3′, antisense: 5′-CAC TTC CTC CAG GAT GTT GT -3′; IL-1β, sense: 5′-CTCCATGAGCTTTGTACAAGG-3′, antisense: 5′-TGCTGATGTACCAGTTGGGG-3′; glyceraldehyde-3-phosphate dehydrogenase, sense: 5′-GGTGAAGGTCGGTGTGAACG-3′, antisense: 5′-TTGGCTCCACCCTTCAAGTG-3′. The expression of iNOS and IL-1β was presented after capturing the gel image with a Fluor Chem 8800 Imaging System (Alpha Innotech Corporation).

### PCR super array analyses of integrin genes

Mouse Integrin Gene Family I and II MultiGene-12 RT-PCR Profiling Kit (SupperArray, PM-003B and PM-004B; SABioscience/Biomol, Hamburg, Germany) profiles the expression of 24 key integrin genes in mouse cells. Briefly, total RNA was isolated in mouse MBP-primed Th1 and Th2 cells using Qiagen RNA isolation kit followed by the synthesis of cDNA as described previously. Next, cDNA samples were diluted by 5 times and then 2 μL of diluted cDNA was added in each well of 12 well array strip followed by the amplification of cDNA using syber green technology in AB7500 standard real time PCR machine. The resulting Ct value was normalized with housekeeping gene GAPDH and then plotted in heat map explorer software.

### Real Time PCR analysis for iNOS and IL-1β mRNA

It was performed using the ABI-Prism7700 sequence detection system (Applied Biosystems) as described earlier [22]. Briefly, reactions were performed in 96-well optical reaction plates on cDNA equivalent to 50 ng of DNase-digested RNA in a volume of 25 μl, containing 12.5 μl of TaqMan Universal Master Mix and optimized concentrations of carboxyfluorescein-labeled probe, forward and reverse primers, following manufacturer’s protocol. All primers and FAM-labeled probes for mouse iNOS, IL-1β, and glyceraldehyde-3-phosphate dehydrogenase were obtained from Applied Biosystems. The mRNA expression of iNOS and IL-1β was normalized to the level of glyceraldehyde-3-phosphate dehydrogenase mRNA. Data were processed by the ABI Sequence Detection System 1.6 software and analyzed by analysis of variance.

### Antisense knock-down of CREB in microglial cells

Selective reductions in CREB levels in the microglial cells were achieved by use of an antisense oligonucleotide strategy as described earlier [3]. The following custom-made oligonucleotide sequences were used: CREB antisense, 59 TGGTCATTTAGTTACCGGTG 39 and CREB missense, 59 GACCTCAGGTAGTCGTCGTT 39 (Invitrogen, Life technologies). The antisense sequence was designed at the translation start site of CREB mRNA to inhibit the translation of CREB protein.

### Assay for NO synthesis

Synthesis of NO was determined by assay of culture supernatants for nitrite, a stable reaction product of NO with molecular oxygen. Briefly, supernatants were centrifuged to remove cells, and 400 μl of each supernatant was allowed to react with 200 μl of Griess reagent [23] and incubated at room temperature for 15 min. The optical density of the assay samples was measured spectrophotometrically at 570 nm. Fresh culture media served as the blank. Nitrite concentrations were calculated from a standard curve derived from the reaction of NaNO_2_ in the assay.

## Results

### Anti-inflammatory role of MBP-primed Th2 cells

**Figure 1:**
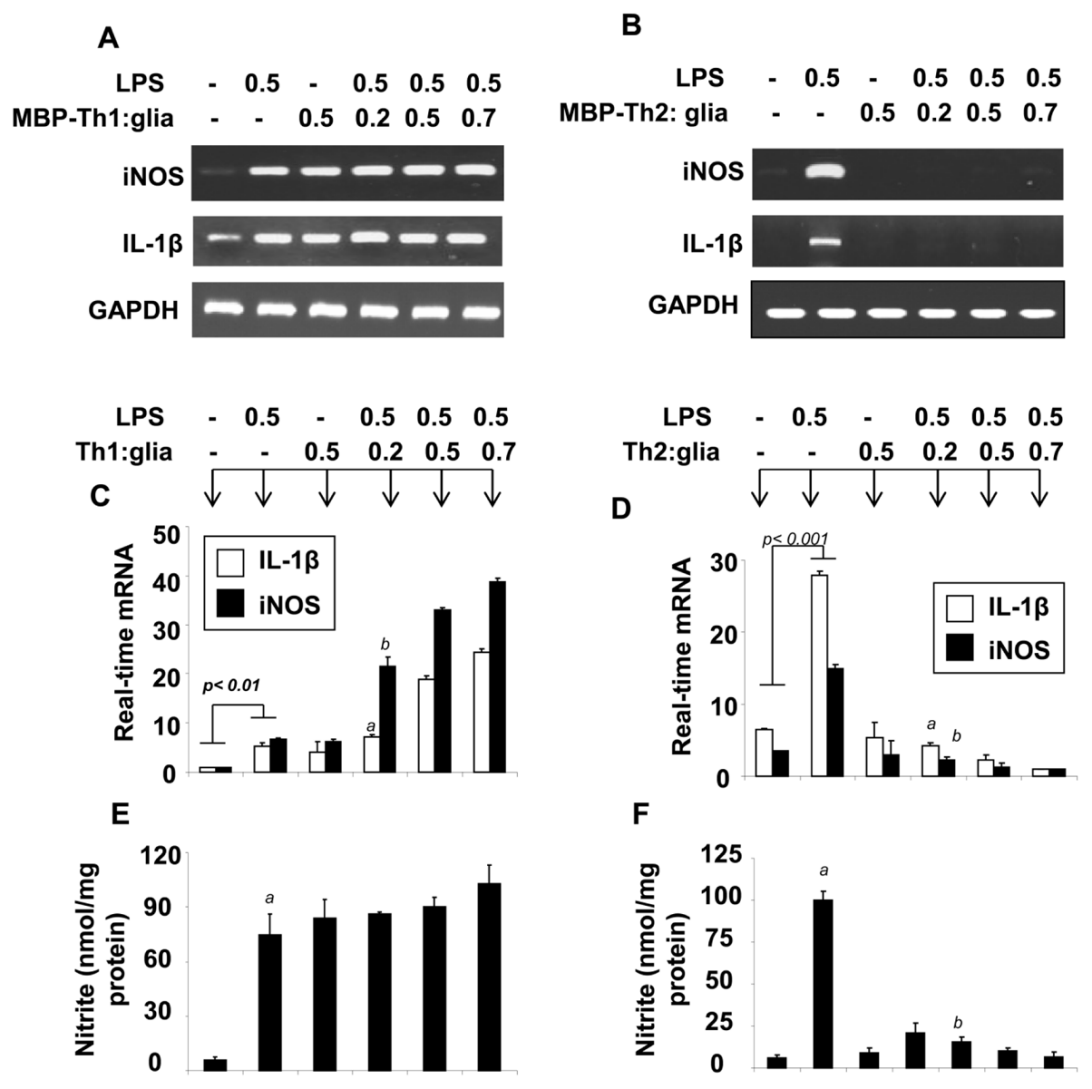
Gemfibrozil treated MBP-primed T cells inhibit iNOS and IL-1β in primary microglia Lymph node cells of MBP-immunized mice were treated with 50 μg/mL MBP alone or together with 25 μM gemfibrozil (Gem) for 4 days to prepare MBP-primed Th1 and Th2 cells respectively. After that, these T cells were cultured over LPS-treated primary microglia in different ratio ranging from 0.2:1 to 0.7:1 for 1 hr followed by the mRNA analysis of iNOS and IL-1β. After 5 hrs of incubation with MBP-primed Th1 cells **(A)** the mRNA expression of iNOS and IL-1β were analyzed in LPS-treated mouse primary microglia by RT-PCR and confirmed by real-time PCR analysis **(C)**. *^a^p*<0.01 vs. control IL-1β; *^b^p*<0.001 *vs*. control iNOS. Similarly, after 5 hrs incubation with Th2 cells the mRNA expression of these genes was performed by RT-PCR **(B)** and then confirmed by quantitative real time PCR analysis **(D)**. *^a^p*<0.0001 vs. IL-1β in LPS-treated microglia; *^b^p*<0.001 *vs*. iNOS in LPS-treated microglia. After 24 hr of stimulation with MBP-primed Th1 **(E)** and Th2 **(F)** cells, supernatants of LPS-treated mouse primary microglia were analyzed for nitrite assay. Results are derived as mean ± SD of three different experiments. *^a^p*<0.0001 *vs*. control nitrite; *^b^p*<0.0001 *vs*. nitrite in LPS-treated cells. Results are mean ± SD of three independent experiments.

LPS is the prototypic inducer of microglial activation [15,24] and MBP-primed Th1 cells have been shown to induce the expression of inflammatory molecules in microglia [25]. Consistently, we also observed that both LPS and Th1 cells were able to induce the mRNA expression of iNOS and IL-1β when treated on primary microglia with their increasing doses. Now we are interested to investigate the effect of MBP primed Th2 cells on the LPS-induced microglial expression of iNOS and IL-1β. Earlier we have shown that gemfibrozil (gem) stimulates the polarization of MBP-primed Th1 cells towards Th2 cells [19] and therefore here we also used gemfibrozil as a Th2 polarizing agent. Here we observed that increasing doses of MBP-primed Th1 cells dose dependently stimulated the expression of iNOS and IL-1β in LPS-stimulated microglia (Figure 1A) indicating the inflammatory properties of MBP-primed Th1 cells. Interestingly, the same LPS failed to induce the mRNA expression of these inflammatory genes in microglia when microglia pre-treated with increasing doses of MBP-primed Th2 cells (Figure 1B). We confirmed our findings by real-time PCR analysis (Figures 1C and 1D). According to quantitative estimation, the LPS-stimulated microglial expression of iNOS and IL-1β gene had been found to be reduced by 5 to 10 fold in the presence of MBP-primed Th2 cells in the ratio of 0.2:1 Th2 cell: microglia. Similarly, MBP-primed Th2 cells also dose-dependently suppressed the LPS-stimulated production of NO microglia (Figures 1E and 1F). These results together suggest that MBP-primed Th2 cells are capable of inhibiting the expression of proinflammatory molecules in LPS-treated mouse primary microglia.

### Autoimmune T cells with Th2 phenotype inhibit microglial inflammatory response

**Figure 2:**
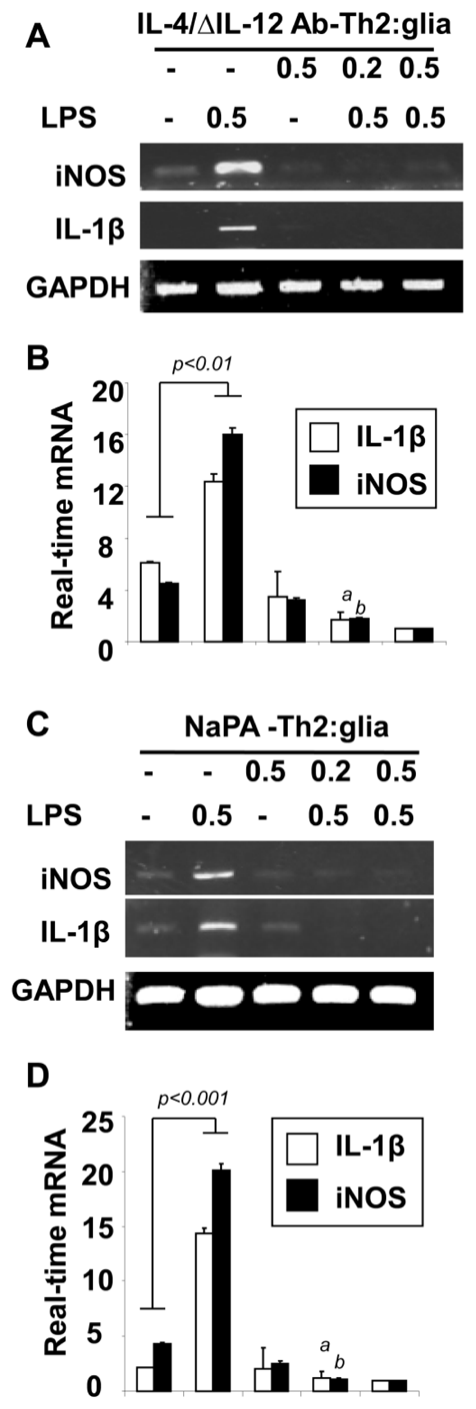
Pre-conditioned MBP-primed Th2 cells suppress iNOS and IL-1β in mouse primary microglia MBP-primed T cells from SJL/J mice were treated with IL-4 plus anti-IL-12 antibody combination (IL-4/ΔIL-12 Ab) and NapA for 4 days to polarize Th2 condition followed by the treatment on LPS-stimulated mouse primary microglia. After 5 hrs of incubation with IL-4/ΔIL-12 Ab, the mRNA expression of iNOS and IL-1β were analyzed by RT-PCR **(A)** and realtime PCR analysis **(B)**. *^a^p*<0.001 *vs*. LPS-stimulated IL-1β; *^b^p*<0.001 *vs*. LPS-stimulated iNOS. After 5 hrs of incubation with NaPA, the mRNA expression of iNOS and IL-1β were analyzed by RT-PCR **(C)** and realtime PCR analysis **(D).** *^a^p*<0.001 *vs*. LPS-stimulated IL-1β; *^b^p*<0.001 *vs*. LPS-stimulated iNOS. Data are mean ± SD of three independent experiments.

Gemfibrozil, a well-known PPAR-α agonist, had been previously shown to produce Th2 cytokines from T cells [26,27]. Therefore, we wanted to investigate whether the anti-inflammatory effect of gemfibrozil treated MBP-primed T cells is due to its Th2 phenotype or due to possible contamination of gemfibrozil itself. To test that, we have treated LPS-stimulated microglial cells with different sets of other MBP-primed Th2 cells. In our previous work, we have demonstrated that in the presence of these treatment conditions, most of the MBP-primed Th1 cells were converted into genuine Th2 cells [3]. These MBP-primed Th2 cells were generated by preconditioning MBP-primed Th1 cells with standard Th2 inducers such as IL-4+anti-IL-12 blocking antibody combination and sodium phenyl acetate (NaPA). Interestingly, (IL-4+anti-IL-12)-treated MBP-primed Th2 cells and NaPA-treated MBP-primed Th2 cells also suppressed the expression of iNOS and IL-1β in microglia (Figures 2A and 2C). Moreover, our realtime PCR analysis further supported that different MBP-primed Th2 cells but not MBP-primed Th1 cells suppressed the LPS-stimulated mRNA expression of inflammatory molecules (Figures 2B and 2D) with their increasing doses.

### MBP-primed Th2 cells suppressed the expression of iNOS and IL-1β in different neurotoxin-stimulated microglia

**Figure 3:**
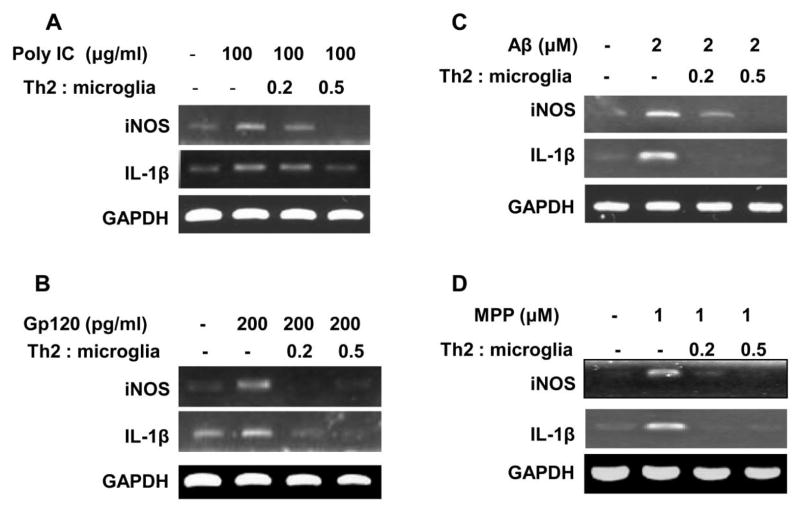
MBP-primed Th2 cells inhibit the expression of iNOS and IL-1β in PolyIC-, gp120-, Aβ-, and MPP+-treated mouse primary microglia Primary microglia received different concentrations (T cells: microglia; 0.2:1, 0.5:1) of gem-treated MBP-primed Th2 cells. After 1 h of contact, culture dishes were shaken and washed three times with HBSS to remove T cells followed by incubation of adherent microglia with 100 μg/ml poly IC, 200 pg/ml gp120, 2 μM fibrillar gp120 and 1μM MPP+. After another 5 hr of incubation, the mRNA expression of IL-β and iNOS was analyzed in **(A)** PolyIC-, **(B)** gp120-, **(C)** Aβ-, and **(D)** MPP+-treated mouse primary microglia.

Microglial activation and inflammation play crucial role in various neuroinflammatory and neurodegenerative diseases including viral encephalopathy, Alzheimer’s disease (AD), Parkinson disease (PD), HIV-associated dementia (HAD), and multiple sclerosis (MS). Therefore, next we were prompted to investigate if MBP-primed Th2 cells were capable of suppressing the expression of inflammatory molecules in microglia in the presence of different neurotoxins such as poly-IC (viral encephalopathy), HIV-1-coat protein gp120 (HAD), Amyloid-β (AD) and MPP+(PD). Surprisingly, our semi-quantitative mRNA analysis revealed that MBP-primed Th2 cells dose-dependently inhibited the expression of inflammatory molecules in different neurotoxin-stimulated microglia (Figures 3A and 3D), suggesting the therapeutic importance of MBP-primed Th2 cells in the suppression of various inflammatory markers that contribute to the pathogenesis of many neuronal diseases.

### MBP-primed Th2 cells suppressed the LPS-stimulated microglial inflammation via-cell-to-cell contact

Next we wanted to investigate the molecular mechanism for the MBP-primed Th2 cell mediated suppression of inflammation in microglia. Earlier we have shown that cell-to-cell contact mechanism between MBP-primed Th2 cells and microglia is crucial for inducing the expression of neurotrophins in microglia [3].

**Figure 4:**
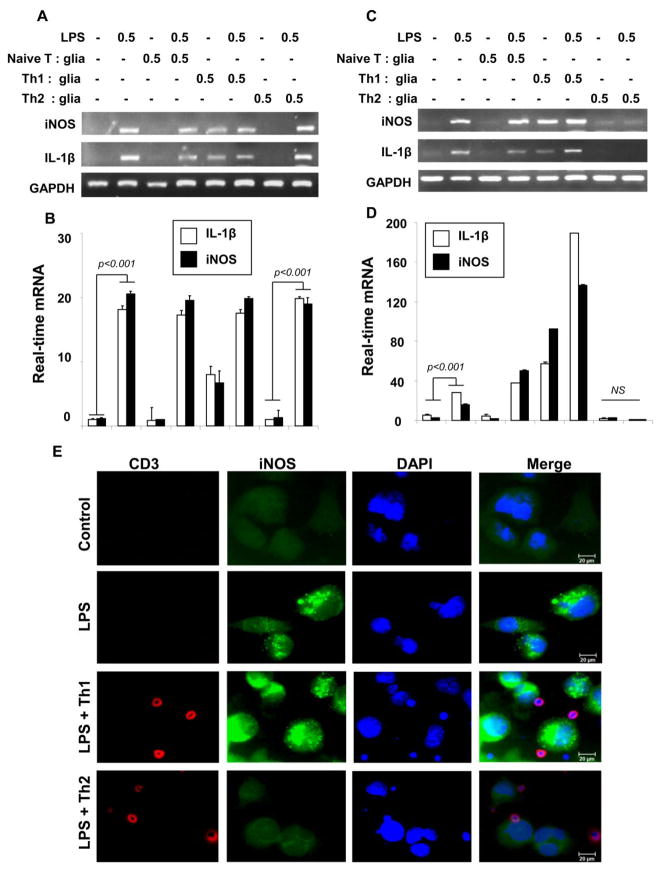
MBP-primed Th2 cells inhibit the LPS-stimulated expression of IL-1β and iNOS in mouse primary microglia via cell-to-cell contact Microglia received different concentrations of Th2 cells within insert. After 1 hr, inserts are removed followed by the stimulation of microglial cells with LPS (0.5 μg/ml) for 1 hr in serum-free condition. After another 5 h of incubation, the mRNA expression of iNOS and IL-1β was analyzed by RT-PCR **(A)** and by real-time PCR **(B)**. Next, Microglia were stimulated by plasma membranes (equivalent to 0.5:1 of T cell: glia) of normal T cells, MBP-primed Th1 and MBP-primed Th2 cells separately. After 1 hr, microglial cells were stimulated with LPS (0.5μg/ml) LPS. After another 5 h, microglial cells were analyzed for the expression of iNOS and IL-1β mRNAs by semi-quantitative RT-PCR **(C)** and quantitative real-time PCR **(D). (E)** Immunocytochemical analyses showed the interaction between CD3-immunostained T cells (red) and iNOS (green) positive LPS-treated microglia. Data are the mean ± S.D. of three different experiments. NS=not significant.

Similarly here we examined if the contact between Th2 cells and glial cells is necessary for the inhibition of iNOS and IL-1β expression. First, Naive T cell, MBP-primed Th1, and Th2 cells were placed in a culture insert at a ratio of 0.5:1 T cell: microglia, where they were in close proximity to, but not contacting LPS-stimulated microglia. Interestingly, we observed almost no suppression of iNOS and IL-1β mRNA expression in microglia when Th2 cells were placed within culture inserts (Figures 4A and 4B). To further prove that it is a contact-mediated effect, we isolated plasma membranes of Th1 and Th2 cells and added to LPS-treated microglia. Plasma membranes of MBP-primed Th2 cells but not Th1 cells significantly attenuated the LPS-stimulated expression of iNOS and IL-1β mRNA (Figures 4C and 4D) whereas membrane of naive T cells does not show any effect in mouse primary microglial cells. On contrary, we observed that membrane fraction of Th1 cells alone or together with LPS stimulated the mRNA expression of iNOS and IL-1β in microglial cells suggesting that T cell-to-microglia contact is also important for Th1 cells to induce the expression of proinflammatory molecules in glial cells (Figures 4C and 4D). Interestingly, our immunocytochemical analyses further revealed that MBP-primed Th2, but not Th1 cells indeed inhibited the LPS-stimulated expression of iNOS protein via cell-to-cell contact mechanism (Figure 4E) in mouse primary microglia. Taken together, our results suggest that MBP-primed Th2 are able to suppress the expression of inflammatory molecules via cell-to-cell contact mechanism.

### Identification of contact molecules on MBP-primed Th2 cells and glia responsible for glial expression of neurotrophins

**Figure 5:**
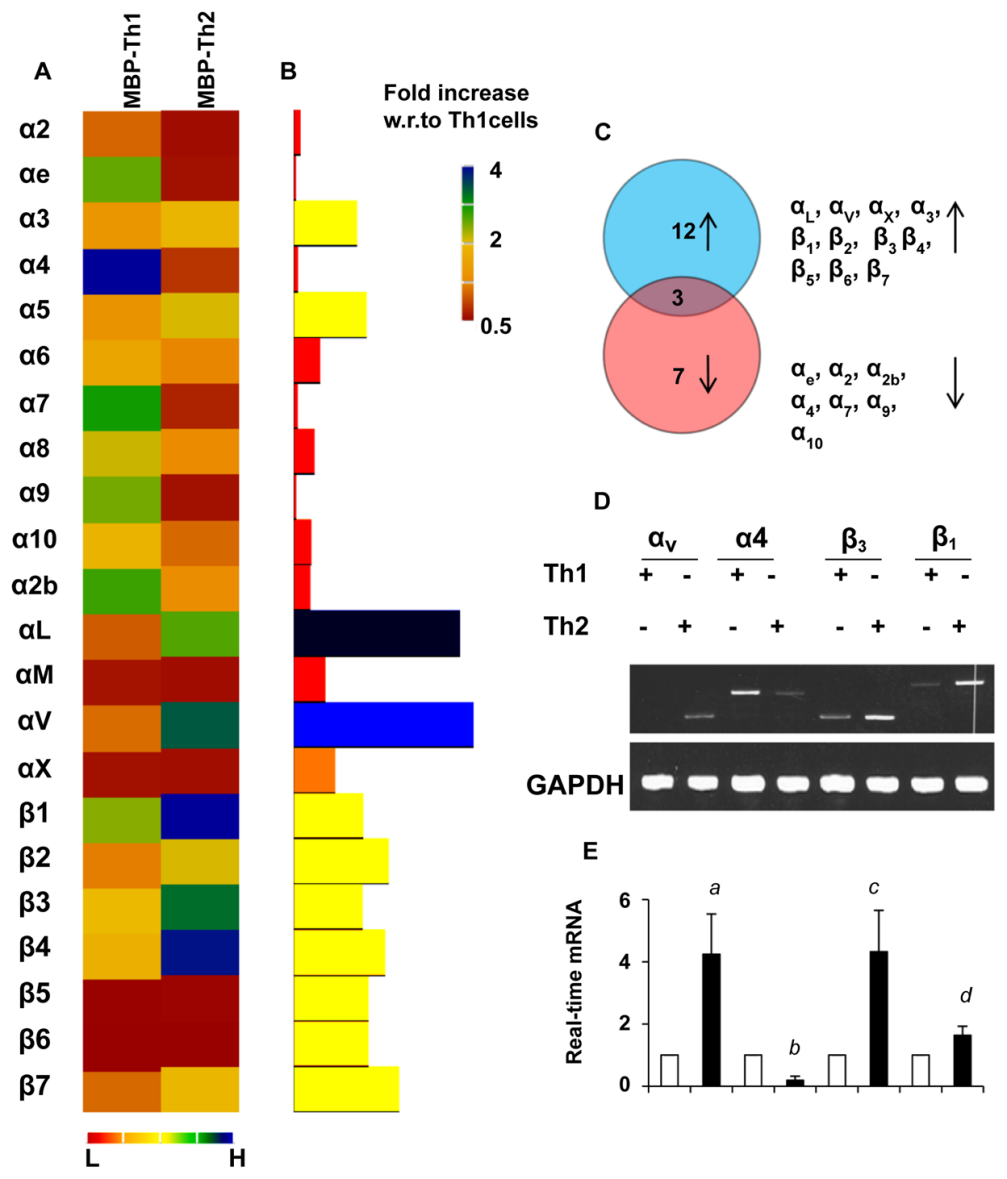
Expression of different integrins in MBP-primed Th1 and Th2 cells Th1 and Th2 cells were analyzed for the expression of different integrins by super array gene array analysis (Super Array). **(A)** Heatmap analysis of differential expression of α and β integrin molecules in Th1 and Th2 cells. **(B)** The histogram analysis of super array. Red indicates the minimum and blue indicates the maximum expression in the scale. **(C)** Venn diagram analysis summarizes the number of integrins upregulated (blue circle), downregulated (red circle), and unaltered (common area) in MBP-primed Th2 cells compared to Th1 cells. The mRNA expression of αV, α4, β1, and β3 in Th1 and Th2 cells was verified by semi-quantitative RT-PCR **(D)** and realtime PCR **(E)** analyses. Results represent three independent experiments. *^a^p*<0.01, *^b^p*<0.05, *^c^p*<0.01, and *^d^p*<0.05 *vs*. control expression of αV, α4, β3, and β1 respectively.

Since T cells express wide range of integrins in the membrane and cell-to-cell contact is very important in our present context of study, we are interested to investigate the expression of different integrin molecules in both MBP-primed Th1 and Th2 cells. Our multi-PCR gene array analysis (Figure 5A) revealed that αL, αV, αX, α3, β1, β2, β3 β4, β5, β6, and β7 genes were upregulated whereas the mRNA expression of αe, α2, α2b, α4, α7, α9, and α10 integrins were downregulated in MBP-primed Th2 cells (Figure 5B). The analysis was summarized as shown in the Venn diagram in (Figure 5C). According to our super array analysis, the mRNA expression αV integrin and its binding partner β3 is significantly higher in MBP-primed Th2 cells. Therefore, next we confirmed their mRNA expressions along with two other integrin molecules α4 and β1 by PCR analysis. Both our semiquantitative RT-PCR (Figure 5D) and real time PCR analyses (Figure 5E) confirmed that the expression of αV and β3, but not α4 and β1, was significantly higher in MBP-primed Th2 cells as compared to MBP-primed Th1 cells indicating their possible role in the cross-talk with microglial membrane molecules.

**Figure 6:**
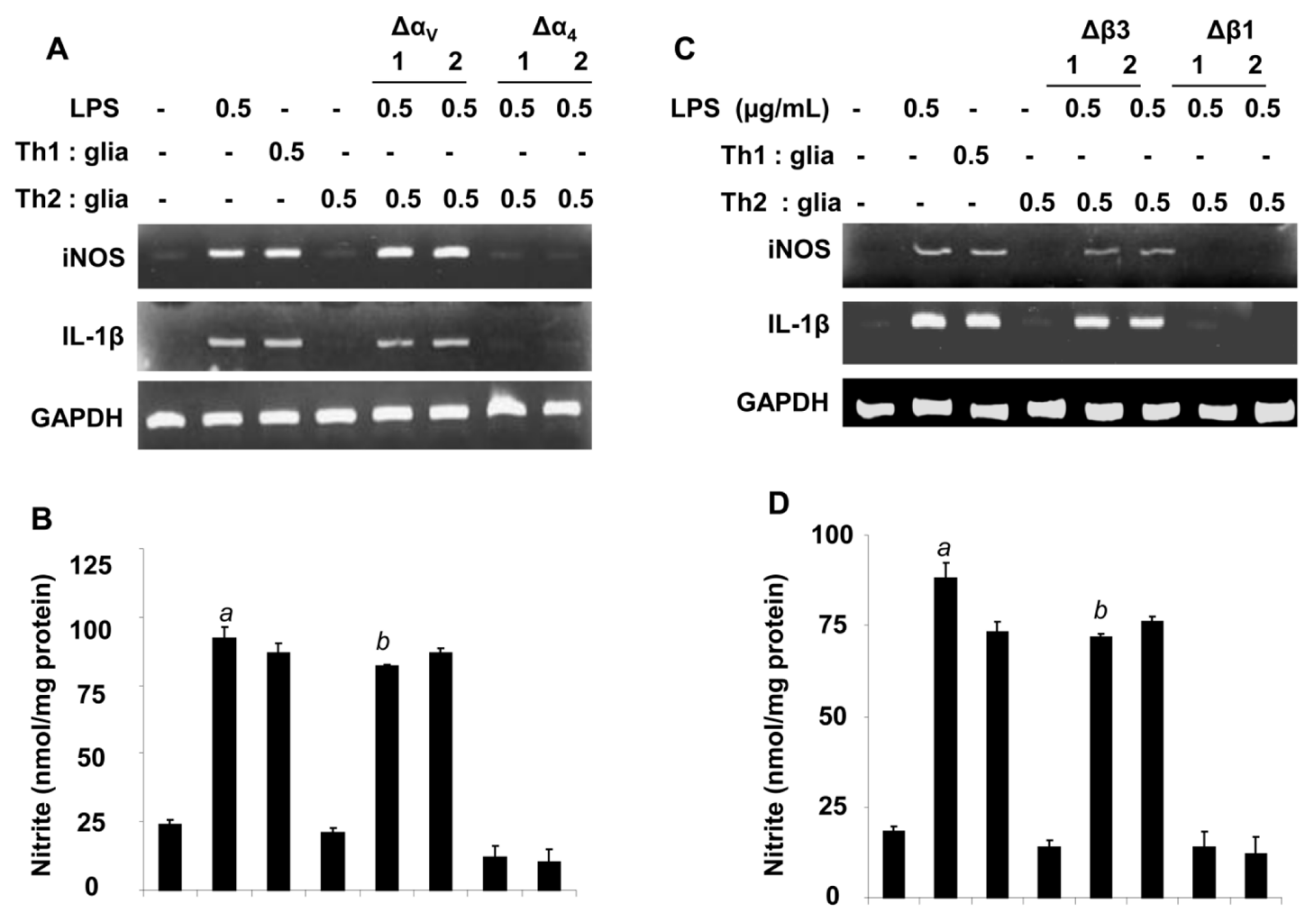
Effect of neutralizing antibodies against α4, αV, β1, and β3 integrins on MBP-primed Th2 cell-mediated inhibition of LPS-stimulated iNOS and IL-1β expression in mouse primary microglia Th2 cells were incubated with different concentrations of neutralizing antibodies against αV, α4, β3, and β1, and then added to mouse primary microglia at a ratio of 0.5:1 T cell: microglia. After 1 h of stimulation, T cells were removed followed by incubation of adherent microglia with LPS (0.5 μg/ml) in serum-free media. (A) After another 5 h of incubation, the expression of iNOS and IL-1β was analyzed by RT-PCR in LPS-stimulated microglial cells incubated with either α4 or αV (1–2 μg/mL) neutralizing antibody-treated Th2 cells. **(B)** After 24 hrs, nitrite was measured in the respective supernatants by Griess method described under “Materials and Methods” section. *^a^p*<0.001 *vs*. nitrite in control glia and *^b^p*<0.001 *vs*. nitrite in only MBP-Th2 treated glia. Similarly, RT-PCR **(C)** analyses of IL-1β and iNOS were performed in microglial cells and nitrite **(D)** production was measured in the supernatants in LPS-stimulated microglia that received β1 or β3 (1–2 μg/mL) blocking antibody-treated Th2 cells. *^a^* <0.001 *vs*. control cells and *^b^p*<0.001 MBP-primed Th2 cells. Data are mean ± S.D. of three different experiments.

Next we investigated whether αV and β3 integrins are involved in the suppression of iNOS and IL-1β in microglia. To study that, we used neutralizing antibodies against α4, αV, β1 and β3. Interestingly neutralization αV and β3, but not α4 and β1 in MBP-primed Th2 cells were unable to suppress the expression of iNOS and IL-1β mRNA expression in LPS-stimulated microglia via cell-to-cell contact mechanism (Figures 6A and 6B). Moreover, we found that application of MBP-primed Th2 cells with increasing concentrations of αV and β3 12 neutralizing antibodies, but not α4 and β1 antibodies, dose dependently induced the mRNA expression of iNOS and IL-1β in LPS-treated microglia (Figures 6A and 6C). In order to confirm our findings we quantified the release of NO in the supernatants of microglial cells incubated with neutralized MBP-primed Th2 cells for 24 hrs. As expected the neutralization of both αV and β3 in Th2 cells was unable to inhibit the release of NO in LPS-treated microglia (Figures 6B and 6D) whereas blocking of α4 and β1. These results together suggest that both of αV and β3 are involved in the suppression of inflammatory molecules in microglia via cell-to-cell contact mechanism.

### The anti-inflammatory role of PDGF receptor β of microglia

**Figure 7:**
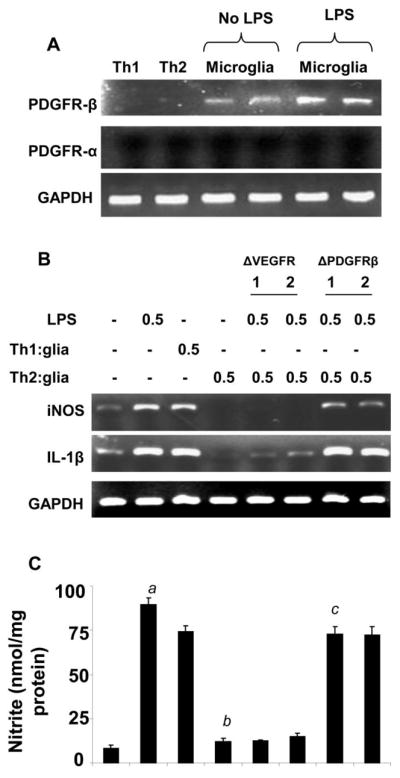
Effect of neutralizing antibodies against PDGFRβ and VEGF-R on MBP-primed Th2 cell-mediated suppression of iNOS and IL-1β in mouse primary microglia **(A)** T cells (MBP-primed Th1 and Th2) and primary microglia (with or without LPS) were analyzed for PDGFRα and PDGFRβ mRNAs by semi-quantitative RT-PCR. Microglia were incubated with different concentrations of neutralizing antibodies against either PDGFRβ or VEGF-R (1–2 μg/mL). Excess antibodies were removed after 1 h of incubation followed by the stimulation of microglia by Th2 cells at a ratio of 0.5:1 T cell: microglia. After 1 h of stimulation, T cells were removed followed by incubation of adherent microglia with LPS (0.5 μg/ml) in serum-free media. After another 5 h of incubation, the expression of iNOS and IL-1β was analyzed by RT-PCR **(B).** After 24 h of incubation (total), supernatants were used to assay nitrite by Griess method described under “Materials and Methods” section **(C).** Data are mean ± S.D. of three different experiments. *^a^p*<0.001 *vs*. control, *^b^p*<0.001 *vs*. Th1-treated, and *^c^p*<0.001 *vs*. Th2-treated microglial cells.

So far, we explored the anti-inflammatory effect of αV and β3 integrins of T cells when allowed to cross-talk with microglia. Next we want to investigate the receptor on microglial cells that interacts with αVβ3 complex of Th2 cells in order to suppress microglial inflammation. It has been reported earlier that αVβ3 integrin complex interacts with PDGF receptor β. Therefore, next we were interested to study if microglia expresses PDGFRβ. Our mRNA analysis (Figure 7A) indicated that primary microglia cells expressed PDGFRβ, but not PDGFRα and that expression was even upregulated when treated with LPS. Next, we wanted to explore the effect of PDGFRβ on the expression of iNOS and IL-1β in LPS-treated microglia when incubated with MBP-primed Th2 cells. Microglia treated with increasing concentrations of PDGFRβ neutralizing antibody, but not VEGFR antibody, was unable to suppress the mRNA expression of iNOS and IL-1β (Figure 7B) and production of nitrite (Figure 7C) in LPS-treated microglia in the presence of MBP-primed Th2 cells suggesting that the interaction between PDGFRβ receptor in microglia and the αVβ3 integrin complex of MBP-primed Th2 cell is crucial in the suppression of inflammatory molecules in mouse microglial cells.

### The DNA binding activity of CREB in MBP-primed T cell mediated expression of IL-1β and iNOS

**Figure 8:**
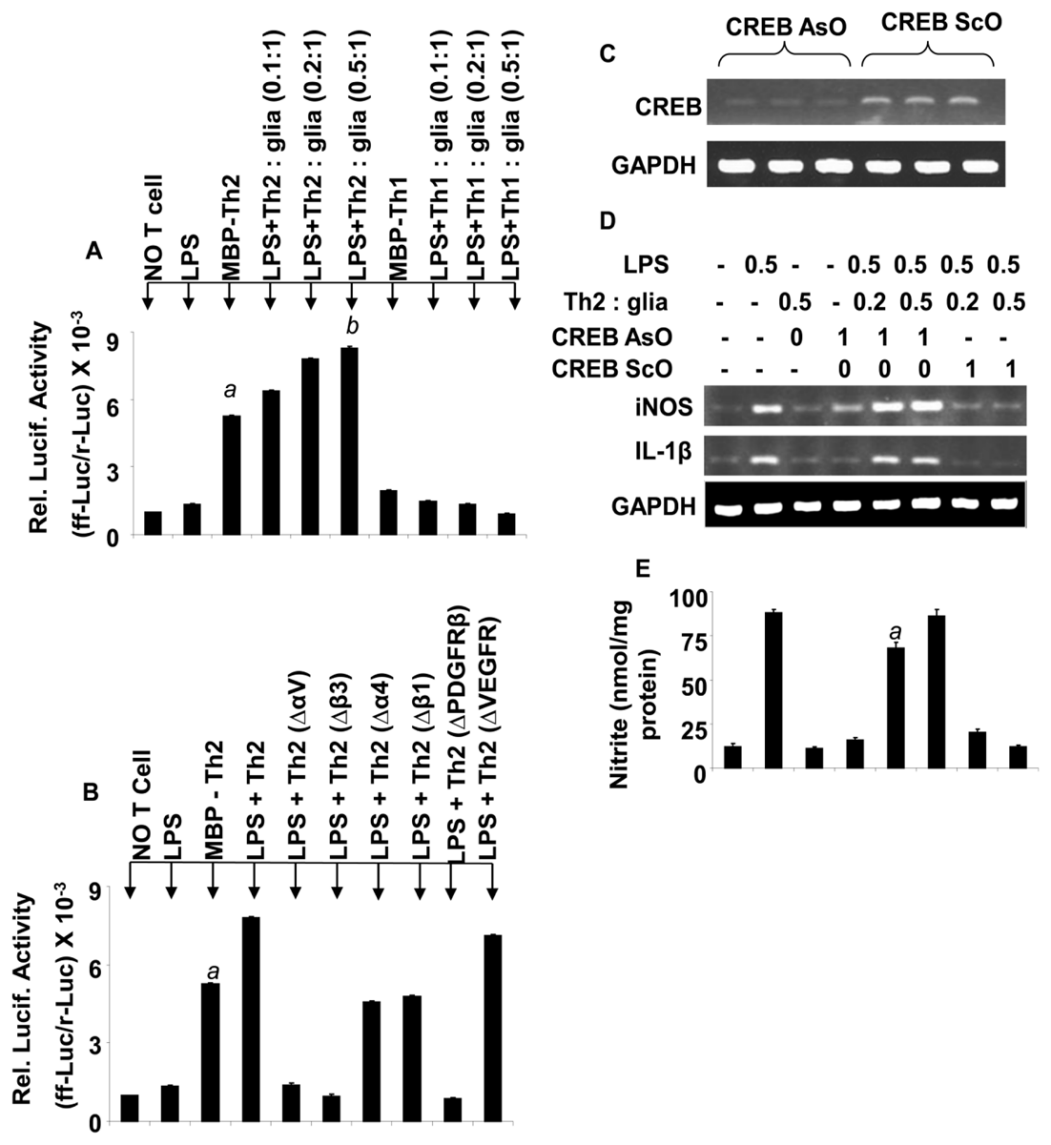
Role of CREB in MBP-primed Th2 cell contact-inhibited glial expression of iNOS and IL-1β **(A)** Mouse BV-2 microglia were co-transfected with pCRE-Luc and pRL-TK. After 24 hr of transfection, cells were stimulated with Th1 and Th2 cells. After 1 hr, T cells were removed and adherent microglia received LPS (0.5 μg/ml) in serum-free media. After 5 hr, firefly and Renilla luciferase activities were assayed. Data are mean ± S.D. of three different experiments. *^a^p*<0.001 *vs*. control (no T cells), *^b^p*<0.01 *vs*. MBP-Th2, and *^c^p*<0.01 *vs*. MBP-Th2 treated cells. **(B)** Th2 cells were treated with 0.5 μg/ml of antibodies against αV (ΔαV), α4 (Δα4), β3, (Δβ3), that were previously transfected with pCRE-Luc and pRL-TK. *^a^p*<0.001 *vs*. control, *^b^p*<0.001 *vs*. LPS+MBP-Th2, and *^c^p*<0.001 *vs*. LPS+MBP-Th2 treated cells. Similarly BV-2 microglia was transfected with pCRE-Luc and pRL-TK. After 24 h of transfection, microglia were incubated with different concentrations of antibodies against either PDGFRβ (ΔPDGFRβ) or VEGF-R (ΔVEGFR) for 1 h followed by stimulation with Th2 cells (0.5:1 of T cell: glia). After 5 h, firefly and Renilla luciferase activities were assayed in microglia. Data are mean ± S.D. of three different experiments. **(C)** Primary microglia was incubated with 1 μM antisense (ASO) and scrambled (ScO) oligonucleotides against CREB. After 42 h of incubation, the expression of CREB mRNA was examined by RT-PCR. **(D)** Microglia preincubated with 1 μM ASO or ScO against CREB for 36 h were stimulated with Th2 cells and LPS as described above. After 5 h, microglia were analyzed for iNOS and IL-1β mRNAs by RT-PCR. **(E)** After 24 h, concentration of nitrite was analyzed in supernatants by ELISA. *^a^p*<0.001 *vs*. nitrite in Th2 -treated microglial cells **(F)** A schematic diagram of Th2 cell and microglial interaction leading to the activation of CREB and suppression of inflammation in microglial cells.

Next we are interested to investigate the intracellular signaling mechanism in microglia when allowed to interact with MBP-primed Th2 cells. Earlier it has been shown that both IL-1β and iNOS gene promoters contain one or more binding site for cAMP-responsive element binding protein (CREB) [28–30] and the upstream activation of CREB is required for the downregulation of iNOS and IL-1β in astrocytes [31]. Therefore, next we hypothesized that CREB is also involved in the downregulation of the proinflammatory genes in microglia once incubated with MBP-primed Th2 cells. To verify that we measured CRE- luciferase activity in microglia in the presence of both MBP-primed Th1 and Th2 cells (Figure 8A). Surprisingly, MBP-primed Th2, but not Th1 cells significantly induced the CRE-luciferase activity in microglia suggesting that the activation of CREB in microglia can be achieved by the contact between MBP-primed Th2 cell and microglia.

Next, we wanted to study if the interaction between αVβ3 integrin complex of Th2 cells and PDGFRβ receptor of microglia is required for the activation of CREB in microglial cells. Interestingly, the neutralization of αV and β3, but not α4 and β1 in Th2 cells inhibited the activation of CREB in microglial cells (Figure 8B). Similarly, the blocking of PDGFRβ, but not VEGFR attenuated the activation of CREB in microglia once allowed to come in contact with MBP-primed Th2 cells (Figure 8B). Next, we wanted to study if the activation CREB is required in the suppression of iNOS and IL-1β in microglia in the presence of MBP-primed Th2 cells. Interestingly, treatment with specific antisense oligonucleotide (Aso) of CREB (Figure 8C), but not scrambled oligonucleotide (ScO) failed to inhibit the mRNA expression of iNOS and IL-1β in microglial cells when treated with MBP-primed Th2 cells, suggesting that the microglial activation of CREB (Figure 8D) is required for the Th2 cell-mediated inhibition of inflammatory genes in microglia. The results had been confirmed by quantitative PCR analysis (Figure 8E).

## Discussion

MBP-primed Th2 cells have been shown to be beneficial in autoimmune diseases like MS [3,32]. However, the mechanism by which Th2 cells suppresses the glial activation is poorly understood. Here we have reported a novel signaling mechanism by which Th2 cell suppresses glial activation. Severe activation of glial cells (microglia and astroglia) or gliosis has been implicated in the pathogenesis of a wide range of neuroinflammatory and neurodegenerative disorders including multiple sclerosis (MS), Alzheimer’s disease (AD), Parkinson’s disease (PD), stroke, Creutzfeld-Jacob disease, and HIV-dementia [33]. Upon activation, glial cells produce and secrete potentially neurotoxic pro-inflammatory molecules including cytokines, reactive oxygen species, and reactive nitrogen species that play an important role in the pathogenesis of neurodegenerative and neuroinflammatory disorders [33–36]. Recently we have shown that MBP-primed Th1 cells induce different proinflammatory molecules (IL-1β, IL-1α, TNF-α, IL-6, and NO) in microglia through cell-to-cell contact [4,5]. Here we demonstrate that MBP-primed Th2 cells suppress the expression of IL-1β and NO in glial cells via cell-to-cell contact. Our conclusion is based on the following observations. First, MBP-primed Th2, but not Th1, cells inhibited the expression of iNOS and IL-1β in LPS-stimulated mouse primary microglia with their increasing doses. Second, MBP-primed Th2 cells also dose-dependently inhibited the expression of iNOS and IL-1β in poly-IC-, gp120-, Aβ-, and MPP+-stimulated mouse primary microglial cells suggesting the beneficial role of Th2 cells in different neurodegenerative disorders. Third, the placement of Th2 cells in a culture insert, where they were in close proximity to, but not contacting microglia, was unable to inhibit the expression of inflammatory molecules in LPS-stimulated microglial cells. Fourth, plasma membranes of MBP-primed Th2 cells but not Th1 cells alone inhibited the expression of iNOS and IL-1β in LPS-stimulated glial cells.

Since Th2 cell-to-microglia attachment is important for the suppression of glial inflammation, identification of the receptor molecule in Th2 cells is mandatory to understand the contact-mediated suppression of inflammatory molecules in microglial cells. Earlier we have shown that α4 and β1 integrin molecules of Th1 cells are inflammatory as these integrins directly stimulate the production of IL-1β and NO in glial cells [37] via similar cell-to-cell contact mechanism. In our present manuscript we have reported that Th2 cells are associated with the upregulated expression of αV and β3 integrin molecules, which are shown to suppress the expression of IL-1β and NO in microglial cells. First, our mRNA based superarray analyses followed by individual PCR reaction confirmed that Th2 cells are associated with the elevated level of αV and β3, but not α4 and β1 integrins. Second, the neutralization of αV and β3 integrins in Th2 cells abrogated their ability to suppress the LPS-stimulated expression of IL-1β and NO in microglial cells.

Platelet derived growth factor receptor beta (PDGFRβ) has been known to be the receptor of αVβ3 integrin molecules [38]. Consistent with previous reports [39], we also observed that microglial cells express significant amount of PDGFRβ, but not PDGFRα. Interestingly, blocking of PDGFRβ, but not other related integrin receptors like VEGFR, was unable to inhibit the LPS-stimulated expression of IL-1β and NO in the presence of Th2 cells suggesting the anti-inflammatory role of PDGFRβ receptor.

Activation of CREB is often observed to be associated with the reduction of inflammatory events in the CNS [40,41]. In our recent study, we have shown that the activation CREB directly regulates the transcription of anti-inflammatory molecule IL-1Ra in neurons, which is an endogenous antagonist ligand of IL-1 receptor [42]. In that study we have shown that the activation of PI3K-Akt pathway is required for the downstream activation of CREB and subsequent transcription of IL-1Ra molecule. In another study, we have shown that PI3K-Akt-CREB pathway is also needed for the suppression of NFkB activation via transcriptional upregulation of IkBα [43]. Since NF-kB is required for the expression of inflammatory molecules in microglial cells, CREB is involved in the suppression of inflammatory molecule via suppression of NFkB activation in microglial cells. Therefore, we were prompted to investigate if CREB was playing a role in T cell contact-mediated suppression of iNOS and IL-1β in microglial cells. Interestingly, only MBP-primed Th2 cells but not Th1 cells induced the activation of CREB in glia by cell-to-cell contact. Furthermore, Th2 cells were unable to suppress the LPS-stimulated expression of iNOS and IL-1β in microglia in which CREB was knocked down by CREB AsO. Interestingly, the neutralization of αV and β3 in Th2 cells abrogated the ability of these cells to induce contact-mediated activation of CREB in glial cells. Similarly, functional blocking of PDGFRβ but not VEGF-R on glial cells also suppressed Th2 cell-induced activation of CREB in glial cells. Therefore our study highlights a novel mechanism of CREB regulation in microglial cells where the cross-talk between αVβ3 integrin complex of Th2 cells and PDGFRβ of microglia is crucial for the downstream activation of CREB.

In summary our present study proposes a novel anti-inflammatory role of Th2 cells owing to T cell-to-microglia contact. Here we demonstrate that αV and β3 integrin molecules in Th2 cells and PDGFRβ receptor in microglial cells play crucial role in conferring the anti-inflammatory properties in microglia via activation of CREB.

## References

[1] Martin R, McFarland HF, McFarlin DE (1992). Immunological aspects of demyelinating diseases. Annu Rev Immunol.

[2] Liu H, MacKenzie-Graham AJ, Palaszynski K, Liva S, Voskuhl RR (2001). “Classic” myelin basic proteins are expressed in lymphoid tissue macrophages. J Neuroimmunol.

[3] Roy A, Liu X, Pahan K (2007). Myelin basic protein-primed T cells induce neurotrophins in glial cells via alphavbeta3 [corrected] integrin. J Biol Chem.

[4] Dasgupta S, Jana M, Liu X, Pahan K (2002). Myelin basic protein-primed T cells induce nitric oxide synthase in microglial cells. Implications for multiple sclerosis. J Biol Chem.

[5] Dasgupta S, Jana M, Liu X, Pahan K (2003). Role of very-late antigen-4 (VLA-4) in myelin basic protein-primed T cell contact-induced expression of proinflammatory cytokines in microglial cells. J Biol Chem.

[6] Benveniste EN (1997). Role of macrophages/microglia in multiple sclerosis and experimental allergic encephalomyelitis. J Mol Med (Berl).

[7] Burguillos MA, Deierborg T, Kavanagh E, Persson A, Hajji N (2011). Caspase signalling controls microglia activation and neurotoxicity. Nature.

[8] Lin MT, Beal MF (2006). Mitochondrial dysfunction and oxidative stress in neurodegenerative diseases. Nature.

[9] Alirezaei M, Kemball CC, Whitton JL (2011). Autophagy, inflammation and neurodegenerative disease. Eur J Neurosci.

[10] Lund S, Porzgen P, Mortensen AL, Hasseldam H, Bozyczko-Coyne D (2005). Inhibition of microglial inflammation by the MLK inhibitor CEP-1347. J Neurochem.

[11] Zassler B, Schermer C, Humpel C (2003). Protein kinase C and phosphoinositol-3-kinase mediate differentiation or proliferation of slice-derived rat microglia. Pharmacology.

[12] Westra J, Doornbos-van der Meer B, de Boer P, van Leeuwen MA, van Rijswijk MH (2004). Strong inhibition of TNF-alpha production and inhibition of IL-8 and COX-2 mRNA expression in monocyte-derived macrophages by RWJ 67657, a p38 mitogen-activated protein kinase (MAPK) inhibitor. Arthritis Res Ther.

[13] Campbell J, Ciesielski CJ, Hunt AE, Horwood NJ, Beech JT (2004). A novel mechanism for TNF-alpha regulation by p38 MAPK: involvement of NF-kappa B with implications for therapy in rheumatoid arthritis. J Immunol.

[14] Jeohn GH, Cooper CL, Jang KJ, Liu B, Lee DS (2002). Gö6976 inhibits LPS-induced microglial TNFalpha release by suppressing p38 MAP kinase activation. Neuroscience.

[15] Roy A, Fung YK, Liu X, Pahan K (2006). Up-regulation of microglial CD11b expression by nitric oxide. J Biol Chem.

[16] Ghosh A, Roy A, Liu X, Kordower JH, Mufson EJ (2007). Selective inhibition of NF-kappaB activation prevents dopaminergic neuronal loss in a mouse model of Parkinson’s disease. Proc Natl Acad Sci U S A.

[17] Mondal S, Roy A, Jana A, Ghosh S, Kordower JH (2012). Testing NF-κB-based therapy in hemiparkinsonian monkeys. J Neuroimmune Pharmacol.

[18] Pahan K, Sheikh FG, Namboodiri AM, Singh I (1997). Lovastatin and phenylacetate inhibit the induction of nitric oxide synthase and cytokines in rat primary astrocytes, microglia, and macrophages. J Clin Invest.

[19] Dasgupta S, Zhou Y, Jana M, Banik NL, Pahan K (2003). Sodium phenylacetate inhibits adoptive transfer of experimental allergic encephalomyelitis in SJL/J mice at multiple steps. J Immunol.

[20] Roy A, Ghosh A, Jana A, Liu X, Brahmachari S (2012). Sodium phenylbutyrate controls neuroinflammatory and antioxidant activities and protects dopaminergic neurons in mouse models of Parkinson’s disease. PLoS One.

[21] van Seventer GA, Semnani RT, Palmer EM, McRae BL, van Seventer JM (1998). Integrins and T helper cell activation. Transplant Proc.

[22] Dasgupta S, Jana M, Zhou Y, Fung YK, Ghosh S (2004). Antineuroinflammatory effect of NF-kappaB essential modifier-binding domain peptides in the adoptive transfer model of experimental allergic encephalomyelitis. J Immunol.

[23] Pahan K, Sheikh FG, Liu X, Hilger S, McKinney M (2001). Induction of nitric-oxide synthase and activation of NF-kappaB by interleukin-12 p40 in microglial cells. J Biol Chem.

[24] Garcion E, Sindji L, Montero-Menei C, Andre C, Brachet P (1998). Expression of inducible nitric oxide synthase during rat brain inflammation: regulation by 1,25-dihydroxyvitamin D3. Glia.

[25] Dasgupta S, Jana M, Liu X, Pahan K (2005). Myelin basic protein-primed T cells of female but not male mice induce nitric-oxide synthase and proinflammatory cytokines in microglia: implications for gender bias in multiple sclerosis. J Biol Chem.

[26] Lovett-Racke AE, Hussain RZ, Northrop S, Choy J, Rocchini A (2004). Peroxisome proliferator-activated receptor alpha agonists as therapy for autoimmune disease. J Immunol.

[27] Racke MK, Gocke AR, Muir M, Diab A, Drew PD (2006). Nuclear receptors and autoimmune disease: the potential of PPAR agonists to treat multiple sclerosis. J Nutr.

[28] Roach SK, Lee SB, Schorey JS (2005). Differential activation of the transcription factor cyclic AMP response element binding protein (CREB) in macrophages following infection with pathogenic and nonpathogenic mycobacteria and role for CREB in tumor necrosis factor alpha production. Infect Immun.

[29] Tsukada J, Saito K, Waterman WR, Webb AC, Auron PE (1994). Transcription factors NF-IL6 and CREB recognize a common essential site in the human prointerleukin 1 beta gene. Mol Cell Biol.

[30] Bhat NR, Feinstein DL, Shen Q, Bhat AN (2002). p38 MAPK-mediated transcriptional activation of inducible nitric oxide synthase in glial cells. Roles of nuclear factors, nuclear factor kappa B, cAMP response element-binding protein, CCAAT/enhancer-binding protein-beta, and activating transcription factor-2. J Biol Chem.

[31] Zhao L, Brinton RD (2004). Suppression of proinflammatory cytokines interleukin-1beta and tumor necrosis factor-alpha in astrocytes by a V1 vasopressin receptor agonist: a cAMP response element-binding protein-dependent mechanism. J Neurosci.

[32] Hendrix S, Nitsch R (2007). The role of T helper cells in neuroprotection and regeneration. J Neuroimmunol.

[33] González-Scarano F, Baltuch G (1999). Microglia as mediators of inflammatory and degenerative diseases. Annu Rev Neurosci.

[34] Maimone D, Gregory S, Arnason BG, Reder AT (1991). Cytokine levels in the cerebrospinal fluid and serum of patients with multiple sclerosis. J Neuroimmunol.

[35] Ruddle NH, Bergman CM, McGrath KM, Lingenheld EG, Grunnet ML (1990). An antibody to lymphotoxin and tumor necrosis factor prevents transfer of experimental allergic encephalomyelitis. J Exp Med.

[36] Samoilova EB, Horton JL, Hilliard B, Liu TS, Chen Y (1998). IL-6-deficient mice are resistant to experimental autoimmune encephalomyelitis: roles of IL-6 in the activation and differentiation of autoreactive T cells. J Immunol.

[37] Brahmachari S, Pahan K (2010). Gender-specific expression of beta1 integrin of VLA-4 in myelin basic protein-primed T cells: implications for gender bias in multiple sclerosis. J Immunol.

[38] Schneller M, Vuori K, Ruoslahti E (1997). Alphavbeta3 integrin associates with activated insulin and PDGFbeta receptors and potentiates the biological activity of PDGF. EMBO J.

[39] Masuda J, Tsuda M, Tozaki-Saitoh H, Inoue K (2009). Intrathecal delivery of PDGF produces tactile allodynia through its receptors in spinal microglia. Mol Pain.

[40] Willis SA, Nisen PD (1995). Inhibition of lipopolysaccharide-induced IL-1 beta transcription by cyclic adenosine monophosphate in human astrocytic cells. J Immunol.

[41] Galea E, Feinstein DL (1999). Regulation of the expression of the inflammatory nitric oxide synthase (NOS2) by cyclic AMP. FASEB J.

[42] Corbett GT, Roy A, Pahan K (2012). Gemfibrozil, a lipid-lowering drug, upregulates IL-1 receptor antagonist in mouse cortical neurons: implications for neuronal self-defense. J Immunol.

[43] Khasnavis S, Jana A, Roy A, Mazumder M, Bhushan B (2012). Suppression of nuclear factor-κB activation and inflammation in microglia by physically modified saline. J Biol Chem.

